# Oral Manifestations in the American Tegumentary Leishmaniasis

**DOI:** 10.1371/journal.pone.0109790

**Published:** 2014-11-11

**Authors:** Daniel Cesar Silva da Costa, Mariana Reuter Palmeiro, João Soares Moreira, Ana Cristina da Costa Martins, Aline Fagundes da Silva, Maria de Fátima Madeira, Leonardo Pereira Quintella, Eliame Mouta Confort, Armando de Oliveira Schubach, Fátima da Conceição Silva, Cláudia Maria Valete-Rosalino

**Affiliations:** 1 Evandro Chagas National Institute of Infectious Diseases, Oswaldo Cruz Foundation, Rio de Janeiro-RJ, Brazil; 2 Laboratory of Immunoparasitology, Oswaldo Cruz Institute, Oswaldo Cruz Foundation, Rio de Janeiro-RJ, Brazil; 3 Department of Otorhinolaryngology and Ophthalmology, Federal University of Rio de Janeiro, Rio de Janeiro-RJ, Brazil; The Ohio State University, United States of America

## Abstract

**Introduction:**

American tegumentary leishmaniasis (ATL) can affect the skin or mucosa (mucocutaneous leishmaniasis – MCL) including the oral cavity. MCL oral lesions are often confused with other oral diseases, delaying diagnosis and specific treatment, and increasing the likelihood of sequelae. Thus, increasing the knowledge of the evolution of ATL oral lesions can facilitate its early diagnosis improving the prognosis of healing.

**Objectives:**

Evaluate the frequency of ATL oral lesion and describe its clinical, laboratory and therapeutic peculiarities.

**Methods:**

A descriptive transversal study was carried out, using data from medical records of 206 patients with MCL examined at the outpatient clinics-IPEC-Fiocruz between 1989 and 2013. Proportions were calculated for the categorical variables and the association among them was assessed by the Pearson's chi-square test. Measures of central tendency and dispersion were used for the continuous variables and their differences were assessed by both parametric (t test) and non parametric (Mann-Whitney) tests. P-values <0.05 were considered as significant.

**Results:**

The most affected site was the nose, followed by the mouth, pharynx and larynx. Seventy eight (37.9%) have oral lesions and the disease presented a lower median of the evolution time than in other mucous sites as well as an increased time to heal. The presence of oral lesion was associated with: the presence of lesions in the other three mucosal sites; a smaller median of the leishmanin skin test values; a longer healing time of the mucosal lesions; a higher recurrence frequency; and a smaller frequency of treatment finishing and healing. When the oral lesion was isolated, it was associated with an age 20 years lower than when the oral lesion was associated with other mucosal sites.

**Conclusion:**

Considering the worst therapy results associated with the presence of oral lesions, we suggest that lesions in this location represent a factor of worse prognosis for MCL.

## Introduction

American tegumentary leishmaniasis (ATL) is a parasitic infectious disease transmitted by protozoa of the *Leishmania* genus, through the bite of a vector (insect) of the *Lutzomyia* genus [1]. Eighty eight countries in the world are affected by leishmaniasis. In the Americas, ATL is distributed from the South of the United States to the South of Argentina [2], [3]. In Brazil (2012) the number of ATL cases was 15.731 and the detection coefficient 11.1 cases/100.000 [4]. The state of Rio de Janeiro is traditionally acknowledged as an ATL endemic area and the infectious agent was identified as *L. (Viannia) braziliensis* [5].

The most common ATL clinical manifestation is the localized cutaneous form which appears as an ulcerated lesion with granular base and raised borders, which might resolve even without treatment. However, some patients infected by *L. (V.) braziliensis* (1 to 10%) develop mucocutaneous leishmaniasis (MCL) which usually presents gradual tissue destruction associated with intense inflammatory response [6]–[8], affecting the upper respiratory and digestive tracts mucosa. The mucosal lesion usually emerges weeks or years after the initial skin lesion has healed, by probable blood spread from the primary focus [9], [10]. However, a mucosal lesion may appear when a skin ulcer is active [11].

The lesions in the mouth are usually associated with nasal involvement, but it is possible to find one or more lesions only in the oral mucosa. These lesions occur more often on the lip and palate, although lesions in the uvula, gums, tonsils and tongue were already identified. They are characterized by ulcerative-vegetative lesion accompanied by coarse granulations. Patients usually complain of pain, dysphagia and odynophagia [12], [13].

Besides the difficulty in identifying parasites, MCL is often mistaken with other benign or malignant lesions that affect the mucosal tissue. Thus, it is necessary to use several methods to confirm diagnosis such as: serology, histopathology, *Leishmania* culture and molecular methods [14], [15]. Isolated or associated, nasal lesions occur at over than 90% of the cases. Consequently, the MCL diagnosis is based on the investigation of nasal lesions and treatment usually starts without oral lesions investigation and diagnosis. For this reason, literature on this subject is scarce, hampering a better understanding and the diagnosis of this type of mucosal lesion.

In this context, the objective of the present study is to evaluate the frequency of ATL oral lesion occurrence and describe their clinical, laboratory and therapeutic peculiarities.

## Methods

This retrospective study was conducted by reviewing the medical records of 206 patients with Mucocutaneous Leishmaniasis monitored at the Ambulatory/Laboratory for Leishmaniasis Surveillance of the Evandro Chagas Clinical Research Institute, Rio de Janeiro between 1989 and 2013. The study was approved by the Ethics in Research Committee of IPEC/FIOCRUZ (CAAE: 09994613.6.0000.5262) after signing a Statement of Commitment by the researchers involved who have undertaken to keep confidential the identity of patients, and the confidentiality and privacy data. All procedures performed in patients followed a protocol of care, which in 2002 was submitted and approved by the Ethics in Research Committee of IPEC/FIOCRUZ (CAAE: 0016.0.009-02), entitled "Study for the systematic assessment of the patients with American Tegumentary Leishmaniasis in Leishmaniasis Reference Center - Institute Evandro Chagas Clinical Research - Fiocruz". Thereafter, all patients sign a Statement of Informed Consent to carry out the clinical protocol and biological sample collection and storage.

ATL diagnosis was established by two or more of the following parameters: consistent epidemiological history, positive reaction in the leishmanin skin test (LST) and the identification of *Leishmania* genus by imprint, culture or histopathologic examination. To assess the presence and location of mucosal alterations of the upper airways tract, all the patients included had been submitted to evaluation of the upper respiratory tracts mucosa by a 30 degrees Karl Storz rigid endoscope, and a 70 degrees Karl Storz rigid videolaryngoscope (Tuttlingen, Germany) and the obtained images were stored through pictures or films.

Socio-demographic (age, gender, occupation and probable infection place), clinical (clinical classification - Table 1 -; symptoms; lesion number, location and characteristics; LST and presence of sequelae), laboratorial (diagnostic methods) and therapeutic (drug used, dose and treatment time) data were collected from the medical records.

**Table 1:** Clinical classification of mucocutaneous leishmaniasis [18].

Concomitant mucosal form	Skin and mucosal lesions occurring at the same time.
Mucosal form of undetermined origin	Mucosal lesions without evidence of previous cutaneous form.
Late mucosal form	Mucosal lesions with evidence of previous cutaneous form.
Contiguous mucosal form	Periorificial facial skin lesions expanding into mucosal area.

Proportions were calculated for the categorical variables. The times of mucosal lesion evolution and treatment did not present normality in the Shapiro-Wilk test, indicating the use of a non-parametric approach. Thus, median (Md) and interquartile range were supplied for those variables (IQR) and for the variable age and LST, mean ± standard deviation were supplied. The association between the categorical variables was assessed by the Pearson's chi-square test. The difference of the values of the continuous variables was assessed by the t test, in the parametric case and by the Mann-Whitney test in the non-parametric cases. P values smaller than 0.05 indicate statistically significant tests. The Statistical Package for Social Sciences (SPSS) version 16.0 (IBM Company) was used for data analysis.

## Results

We studied 206 patients with MCL, with a mean age of 51.72±17.61 years, with 72.8% males, 85.4% having acquired the infection in Brazilian Southern region and 24.6% farmworkers.

Table 2 shows the clinical and therapeutic peculiarities of the 206 patients with MCL. Concomitant mucosal was the most frequent form. The most affected site was the nose, followed by the mouth, pharynx and larynx. The oral lesions presented a lower median of the evolution time than in other mucosal sites as well as an increased time to heal. One hundred sixty-four patients (79.6%) completed treatment and 130 (63.1%) were followed up to one year, when 121 (93%) were healed and 9 (7%) presented recurrence.

**Table 2:** Clinical and therapeutic characteristics of 206 patients with mucocutaneous leishmaniasis. INI FIOCRUZ, 2014.

Variables		
Clinical classification		
Concomitant mucosal form	78(n)	37.9%
Mucosal form of undetermined origin	55(n)	26.7%
Late mucosal form	52(n)	25.2%
Contiguous mucosal form	21(n)	10.2%
Affected sites		
Nasal cavity	188	91,3%
Oral cavity	78	37,9%
Pharynx	64	31,4%
Larynx	62	30,1%
One site affected	104(n)	50,5%
Nasal cavity	92(n)	44.7%
Oral cavity	7(n)	3.4%
Larynx	5(n)	2.4%
Two sites affected	42(n)	20.5%
Three sites affected	36(n)	17.5%
Four sites affected	24(n)	11.7%
Time of mucosal lesions evolution before diagnosis (months)	24 Md	6–60 IQR
Nasal cavity	24 Md	6–60 IQR
Oral cavity	8Md	3–29 IQR
Pharynx	12 Md	6–26,5 IQR
Larynx	12 Md	6–36 IQR
Treatment		
Meglumine antimoniate	193	93,7%
5 mgSb^5+^/kg/day	165	86,4%
10–20 mgSb^5+^/kg/day	26	13,6%
Amphotericin B	7	3,4%
Other drugs	6	2,9%
Healing time of the mucous lesions (days)	98 Md	60–178 IQR
Nasal cavity	90 Md	58–155,25 IQR
Oral cavity	113 Md	59–205 IQR
Pharynx	91 Md	59,5–172,25 IQR
Larynx	78,5 Md	55,25–223 IQR

n-valid numbers, Md - median, IQR - interquartile range.

From the 206 patients with MCL, 78 (37.9%) patients have oral lesions, with a mean age of 50.2±17.19 years and 23.1% were female and 76.9% male. Table 3 shows the clinical, laboratory and therapeutic peculiarities of the 78 patients with oral lesions. Among them, 71 patients (91%) had oral lesion associated with lesions at other mucosal site and 7(9%) did not present associated lesions. The number of lesions in the oral cavity varied from 1 to 4 (median  = 1). The LST and serology were positive in most of the patients (respectively 97,1% and 75,7%). Culture and imprint were more frequently positive than the histopathology (Table 3). The histopathological examinations detected cellular infiltrate in all oral lesions. In addition, 12.8% presented granules, ulcer and/or tissue destruction. Of the total 78 patients, seven (9%) had already received previous treatment: five with meglumine antimoniate, one with amphotericin B and the other with unknown treatment. Of 57 patients followed up to one year, 48 (84.2%) were healed.

**Table 3:** Clinical, laboratory and therapeutic characteristics of 78 patients with oral leishmaniasis lesions. INI FIOCRUZ, 2014.

Variables		
Clinical classification		
Oral lesions	78 (n)	100%
Concomitant mucosal form	34(n)	43,6%
Mucosal form of undetermined origin	18 (n)	23,1%
Late mucosal form	18(n)	23,1%
Contiguous mucosal form	8(n)	10,2%
Oral lesions associated with other mucosal site lesion	71(n)	91%
Concomitant mucosal form	30(n)	42,3%
Mucosal form of undetermined origin	18(n)	25,4%
Late mucosal form	18(n)	25,4%
Contiguous mucosal form	5(n)	7%
Isolated Oral lesions	7(n)	9%
Concomitant mucosal form	4(n)	57,1%
Contiguous mucosal form	3(n)	42,9%
Leishmanin skin test (N = 66)	27,36(*X*)	±19,63(*S*)
Serology (N = 66)		
Positive	50 (n)	75,7%
Negative	16 (n)	24,3%
Histopathology (N = 45)		
Presence of amastigote forms	24(n)	53,3%
*Imprint* (N = 13)		
Positive	9(n)	69,2%
Negative	4(n)	30,8%
Culture (N = 34)		
Positive	22(n)	64,7%
Negative	5(n)	14,7%
Contaminated	7(n)	20,6%
Treatment		
Meglumine antimoniate	71(n)	91%
5 mgSb^5+^/kg/day	58(n)	82,9%
10 mgSb^5+^/kg/day	4(n)	5,7%
15 mgSb^5+^/kg/day	5(n)	7,1%
20 mgSb^5+^/kg/day	3(n)	4,3%
Amphotericin B	4(n)	5,2%
Not follow treatment	3(n)	3,8%

N- total number, n-valid numbers, *X* - mean, *S* - standard desviation.

Twenty-three patients presented oral sequelae (destruction of anatomical structures) after completing treatment, with the uvula as the most frequently affected site in 19 patients, followed by the soft palate in 14 patients.

The clinical, laboratory and therapeutic characteristics of 206 patients with mucocutaneous leishmaniasis was compared as regard the presence or not of oral lesion, and the results are shown in Table 4. The presence of oral lesion was associated with the presence of lesions in the other three upper respiratory mucosal anatomic sites (Nasal p = 0.008; Larynx p = 0.003 and Pharynx p<0.001), as well as with a greater recurrence frequency (p = 0.003) and smaller occurrence of treatment finishing (p<0.001) and healing (p<0,001). We also observed association of the presence of oral lesion and a smaller median value of the LST diameter (p = 0.015), shorter time of mucosal lesion evolution (p = 0.007) and longer time necessary for healing the mucosal lesion in any site (p<0.001) as well as when only the nasal lesion is considered (p = 0.029).

**Table 4:** Comparison of the presence or not of oral lesion with clinical, laboratory and therapeutic characteristics of 206 patientes with mucocutaneous leishmaniasis. INI FIOCRUZ, 2014.

	Presence of Oral Lesion				
	Yes (N = 78)		No (N = 128)		
Variables	n		n		*p*
Male	60	76,9%	90	70,3%	0,301
Presence of Cutaneous Lesion	42	53,8%	56	43,8%	0,159
Presence of Cutaneous Scar	23	29,5%	46	35,9%	0,341
Presence of Nasal Lesion	66	84,6%	122	95,3%	**0,008**
Presence of Laryngeal Lesion	33	42,3%	29	22,7%	**0,003**
Presence of Pharyngeal Lesion	46	59,0%	18	14,3%	**<0,001**
Recurrence	21	30,9%	14	12,6%	**0,003**
Treatment Finishing	53	70,7%	111	91,0%	**<0,001**
Healing after one year	48	84,2%	82	98,8%	**0,001**
Mucosal Lesion Evolution (months)	73	12 Md	101	24 Md	**0,007**
LST (major diameter)	66	21 Md	109	30 Md	**0,015**
HT of Mucosal Lesions (days)	61	125 Md	106	80,50 Md	**<0,001**
HT of Nasal Lesions (days)	54	116,5 Md	100	80,50 Md	**0,029**
HT of Laryngeal Lesions (days)	25	77 Md	23	80 Md	0,773
HT of Pharyngeal Lesion (days)	38	91 Md	10	96 Md	0,812
Treatment Duration (days)	75	51 Md	123	41 Md	0,217

N- total number, n-valid numbers, Md - median, **bold- significant p value**.

Some clinical, laboratory and therapeutic characteristics of 78 patients with oral leishmaniasis was compared as regard the presence or not of isolated oral lesions, and the results are shown in Table 5. We verified an association between isolated oral lesion and a shorter time of mucosal lesion evolution (p = 0.03) and smaller LST (p = 0.025). Regarding age, a significant difference (p = 0.002) was found when the two groups were compared (isolated oral lesion  = 31.71±10.42 years and oral lesions in the presence of other associated mucosal lesions  = 51.93±16.69 years).

**Table 5:** Evolution of the mucosal lesions, leishmanin skin test and healing times of 78 patients with oral leishmaniasis compared as regard the presence or not of isolated oral lesions by the Mann-Whitney test with p value. INI-FIOCRUZ, 2014.

	Presence of Isolated Oral Lesion				
	Yes (N = 7)		No (N = 71)		
Variables	n	*Median*	n	*Median*	*p*
Mucosal Lesion Evolution (months)	7	4	66	20	**0,03**
LST (major diameter)	6	14	60	22,5	**0,025**
HT of mucosal lesions (days)	6	116,5	55	135	0,934
HT of Oral Lesions (days)	6	116,5	57	109	0,456
Treatment Duration (days)	7	66	68	50,5	0,623

N- total number, n-valid numbers, LST - leishmanin skin test, HT- healing time, **bold- significant p value**.

Table 6 shows some clinical, laboratory and therapeutic characteristics of 206 patients with mucocutaneous leishmaniasis compared as regard the presence or not of isolated nasal lesions. The presence of isolated nasal lesion showed either evolution and healing times inversely related to those observed in the oral lesion. We observed association between isolated nasal lesion and a longer time of mucosal lesion evolution (p = 0.024), a shorter time for mucosal lesion healing (p<0.001) and shorter time for nasal lesion healing (p = 0.007).

**Table 6:** Evolution of the mucosal lesions, leishmanin skin test and healing times of 206 patients with mucocutaneous leishmaniasis compared as regard the presence or not of isolated nasal lesions by the Mann-Whitney test with p value.

	Presence of Isolated Nasal Lesion				
	Yes (N = 92)		No (N = 114)		
Variables	N	*Median*	n	*Median*	*p*
Mucosal Lesion Evolution (months)	77	24	97	12	**0,024**
LST (major diameter)	80	30	95	24	0,140
HT of mucosal lesions (days)	78	75,5	89	120	**<0,001**
HT of Nasal Lesions (days)	78	75,5	76	105,5	**0,007**
Treatment Duration (days)	88	36,5	110	50	0,110

INI-FIOCRUZ, 2014. N- total number, n-valid numbers, LST - leishmanin skin test, HT- healing time, **bold- significant p value**.

The healing time was longer in the presence of laryngeal lesion (median  = 121.5 days) than in its absence (median  = 92 days) (p = 0.020). Presence or absence of pharyngeal lesions did not show any significant difference in the parameters analyzed.

## Discussion

When we evaluated 78 patients with oral leishmaniasis we observed that this mucosal site was the second most frequently affected by the disease, and was associated with lesions in other upper respiratory tract mucosal sites, a shorter evolution time and factors of worse prognosis such as: longer healing time, higher recurrence frequency and smaller frequency of treatment finishing and healing. When the oral lesion was isolated, it was associated with an age 20 years lower than when the oral lesion was associated with other mucosal sites.

In accordance with the literature, our patients' group with MCL were majority composed by males aged 50 years or more. [13], [16]–[18] However, we identified that the concomitant mucosal form had the highest prevalence, followed by the mucosal form of undetermined origin and the late mucosal form, which is considered by the literature [19], [20] as the most prevalent, was only third in occurrence. This difference might be related to the systematic examination conducted on our service, where all the patients suspected of ATL were examined by a multidisciplinary team which included dermatologists, infectious disease specialists, otorhinolaryngologists and dentists. Regardless of the complaints, the patients were submitted to upper respiratory tracts endoscopy allowing an early diagnosis of the mucosal lesions. The conduct of mucosa systematic examination recommended in 1922 by Klotz and Lindenberg, is rarely followed, remaining restricted to punctual initiatives, such as the case of Boaventura et al. (2006) [16] where the concomitant mucosal lesion was observed in 2.7% cutaneous leishmaniasis (CL) cases [16].

Taking into consideration that the oral cavity have a shorter evolution time than the other mucosal sites, the systematic examination might have favored the identification of the mouth as the second most frequent site. Most studies do not even mention the oral cavity as a location, merely describing isolated regions of the mouth such as palate and lips or associating it with the pharynx [10]–[19]. In addition, the association with conditions that favor local inflammation and infection such as poor oral hygiene [21] might mask the ATL lesions, thus explaining the small frequency of oral involvement reported in literature.

Isolated nasal cavity lesions showed the greatest time of evolution, suggesting that the nose can singly evolve for a long time without requiring medical attention. We suggest that the location of the lesions in the oral cavity may lead to greater discomfort of the patient who would seek for medical care earlier. It is possible that the association of lesions at nasal cavity with other upper respiratory tract sites is not necessarily the natural history of the disease, but a more severe outcome in certain patients [10], [22], [23].

Additionally, the age of the patients with isolated oral lesions was 20 years lower than the observed in patients with lesions in other sites, which may be due to the fact that isolated oral lesions were found in patients who sought medical attention for investigating active cutaneous lesions and not with mucosal lesions at a later stage evolution. It is possible that mucosal lesions, at least in some cases, have insidious and early onset, just passing unnoticed and being diagnosed as CL, only being later mistakenly diagnosed as mucosal late form.

Regarding the diagnosis of the oral lesion, we could not find in literature studies describing results obtained exclusively in this site. However, when we compare it with the general diagnosis of MCL diagnosis, we observe similarity of isolation rates in culture, although a higher detection of amastigotes in the histopathologic examination and imprint. [24], [25] ELISA showed reactivity higher than 90% as mentioned in literature. [26] It was already reported that lesions with shorter evolution time have more parasites [27] suggesting that the early diagnosis carried out at our service might have favored the number of positive exams; in addition it is possible that the oral lesion presents a more significant parasitism due to local inflammatory and not specific infectious processes [21] that favor a influx of parasites through spread by the blood system and the higher number of potentially host cells of the parasite.

The median of the LST values in patients with oral lesion was 9 mm smaller than of those without oral lesion. Additionally, despite the shorter evolution time of oral mucosal lesions until their diagnosis, patients that had lesions in this location, presented longer times for general mucosal and nasal mucosal healing, 45 and 36 days respectively than the patients without oral lesion. In addition, the presence of oral lesion was associated with a higher number of recurrences and a smaller frequency of treatment finishing and healing up to one year after treatment. The occurrence of therapy failure in CL was associated with a short time of lesion evolution (less than 2 months), before the beginning of treatment, and a smaller LST intensity, which could reflect a weak cell immune response. [28] The presence of oral lesion in ATL was also associated with a reduction of food intake, with consequent malnutrition and longer time for lesion healing. [29]

And, finally, the use of small doses of meglumine antimoniate, different from what is indicated in literature [18] proved to be efficient in MCL patient treatment including the oral location.

As conclusion, our results suggest that oral involvement in ATL is associated with worse therapeutic results and may be considered as a factor of worse prognosis in its mucosal form.
