# Correction: Oral Manifestations in the American Tegumentary Leishmaniasis

**DOI:** 10.1371/journal.pone.0117681

**Published:** 2015-01-30

**Authors:** The PLOS ONE Staff

In the Funding section, one grant number from the funder Papes VI/FIOCRUZ/Brazilian National Council for Scientific and Technological Development (CNPQ) is missing. The grant number is: 407582/2012–6.
